# Recent Achievements on Grating Fabrications in Polymer Optical Fibers with Photosensitive Dopants: A Review

**DOI:** 10.3390/polym14020273

**Published:** 2022-01-10

**Authors:** Jie Jiang, Nan Zhang, Rui Min, Xin Cheng, Hang Qu, Xuehao Hu

**Affiliations:** 1 Department of Physics, College of Science, Shantou University, Research Center for Advanced Optics and Photoelectronics, Shantou 515063, China; 18jjiang@stu.edu.cn (J.J.); 18nzhang4@stu.edu.cn (N.Z.); haqux@stu.edu.cn (H.Q.); 2 Key Laboratory of Intelligent Manufacturing Technology of MOE, Shantou University, Shantou 515063, China; 3 State Key Laboratory of Cognitive Neuroscience and Learning, Beijing Normal University at Zhuhai, Center for Cognition and Neuroergonomics, Zhuhai 519087, China; ruimin@bnu.edu.cn; 4 Department of Electrical Engineering, Photonics Research Centre, The Hong Kong Polytechnic University, Kowloon, Hong Kong SAR 997700, China; eechengx@polyu.edu.hk

**Keywords:** polymer optical fiber, dopant, photosensitive, grating, poly-(methyl methacrylate) (PMMA), inscription

## Abstract

This review discusses recent achievements on grating fabrications in polymer optical fibers doped with photosensitive materials. First, different photosensitive dopants in polymer optical fibers (POFs) are summarized, and their refractive index change mechanisms are discussed. Then, several different doping methods to fabricate the photosensitive POFs are presented. Following that, the principles of gratings, including standard fiber Bragg gratings (FBGs), tilted fiber Bragg gratings (TFBGs), chirped fiber Bragg gratings (CFBGs), phase-shifted fiber Bragg gratings (PSFBGs), and long period fiber gratings (LPFGs), are reported. Finally, fabrications of different gratings based on photosensitive POFs in the last 20 years are reported. We present our article clearly and logically, so that it will be helpful for researchers to explore a broad perspective on this proposed topic. Overall, the content provides a comprehensive overview of photosensitive POF fabrications and grating inscriptions in photosensitive POFs, including previous breakthroughs and recent advancements.

## 1. Introduction

Compared with traditional silica optical fibers, polymer optical fibers (POFs) have similar advantages, such as small size, immunity to electromagnetic interference, and multiplexing capabilities, though transmission losses of POFs are higher compared to their counterparts. To date, POFs have been utilized for short-distance data transmission, such as home network connections and automotive applications [1]. Due to unique advantages, such as low Young’s modulus, large negative thermo-optic coefficients, high elastic strain limits, and high bending flexibility, POFs are used for sensing applications of temperature, strain, structural health monitoring, etc. [2,3]. Among all the POF materials, poly- (methyl methacrylate) (PMMA) is the most popular one. Doped PMMA POFs normally demonstrate biocompatible performance and have already been used for in vitro sensing applications, such as the monitoring of human physiological signs and body functions [4,5]. However, due to the limit performance of the PMMA material, doped PMMA POFs are not biodegradable. From this point of view, the investigation of biodegradable POFs based on biodegradable materials remains promising [6,7]. Since the first fiber Bragg grating (FBG) was successfully inscribed in a single-mode (SM) step-index (SI) POF in 1999 [8], grating-based sensors, including standard FBGs, tilted fiber Bragg gratings (TFBGs), chirped fiber Bragg gratings (CFBGs), phase-shifted fiber Bragg gratings (PSFBGs), and long period fiber gratings (LPFGs), have been widely investigated in POFs. For non-doped POFs, the FBG inscription efficiency is very low. For example, in 2002, Liu et al., inscribed Bragg gratings in PMMA-based POFs with ~100% reflectivity by using a 325 nm UV laser, but a very long irradiation time 85 min was taken to achieve that [9]. In 2005, Dobb et al., first demonstrated the inscription of FBGs on an endlessly SM microstructured POF (mPOF), after a UV irradiation of 1 h, and only 10% reflectivity was obtained [10]. In 2014, Sáez-Rodríguez et al., successfully fabricated FBGs with ∼100% reflectivity in undoped PMMA mPOFs via a 325 nm He-Cd CW laser; nevertheless, the inscription process spent more than 2 h [11]. To obtain high quality devices, large refractive index modifications in short time is crucial. Thus, researchers relied on femtosecond laser direct writing technique. In 2012, Alessio et al., achieved FBGs in 2.5 s in a specialty mPOF using an 800 nm femtosecond laser and point-by-point (PbP) technique. However, this required sophisticated regulation technique to focus the pulsed laser on the side of the fiber without holes [12]. In 2021, Cheng et al., demonstrated an optimized 520 nm femtosecond laser PbP inscription system to quickly inscribe FBGs on a novel rectangular SM SI POF [13]. In 2015 and 2018, researchers from Cyprus University of Technology successfully inscribed FBGs with reflectivity of ~70% in commercial gradient-index cyclic transparent optical polymer (CYTOP) perfluorinated fibers with a femtosecond laser operating at 517 nm and line-by-line (LbL) [14] and plane-by-plane (Pl-b-Pl) techniques [15], respectively. Though the femtosecond laser direct writing system is very powerful for FBG inscriptions, it is very complicated and expensive. On the contrary, gratings on the POFs doped with photosensitive materials have been inscribed cost-effectively thanks to the phase mask technique, and consequently mass high-quality grating-based devices can be fabricated in a short time. Until now, there are various refractive index change mechanisms in doped POFs, such as photodegradation [4,16,17,18], photopolymerization [19,20,21,22,23], photoisomerization [24,25,26,27,28,29], and photo-crosslinking [30]. Motivated by this desire, we will present an extensive review on fabrications of photosensitive POFs and fiber gratings.

This review is structured as follows. Photosensitive materials doped in POFs are reported in Section 2. Section 3 focuses on the doping methods for the fabrication of photosensitive POFs. Section 4 presents principles of gratings including FBGs, TFBGs, CFBGs, PSFBGs, and LPFGs. Section 5 introduces grating fabrication techniques based on photosensitive POFs. Finally, Section 6 summarizes the main conclusions and outlines the promising research fields in the future.

## 2. Photosensitive Dopants in POFs

To date, for the improvement of grating quality and inscription efficiency, several kinds of photosensitive materials have been doped in either cores or claddings of PMMA-based POFs. The photosensitive materials as dopants include benzyl dimethyl ketal (BDK) [19,23,31,32,33,34,35], trans-4-stilbenemethanol (TS) [27,28,29,36,37,38], diphenyl disulphide (DPDS) [4,18], 9-vinylanthracene (9-VA) [30], methyl vinyl ketone (MVK) [16], and azobenzene (AZO) [39,40,41,42], etc., whose molecular structures are listed in Table 1. For POFs with doped cores, the dopants normally play two roles, including the enhancement of photosensitivity and increase of core refractive index of the fiber [4,16,19,21,22,23,30,33,39,41,43].

### 2.1. BDK

**Figure 1:**
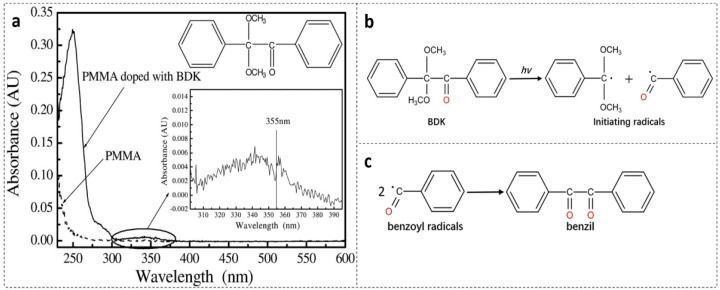
(**a**) Absorption spectra of PMMA and BDK-doped PMMA, the inset shows the zoomed-in absorption spectrum from 302 to 395 nm. Reprinted with permission from [19]. (**b**) Structure change of BDK under UV irradiation [20,21]; (**c**) Formation of benzil molecules from benzoyl radicals generated under UV irradiation on BDK [20,21].

In 2010, Luo et al., introduced BDK as a dopant to PMMA [19], which is a typical photosensitive dye with a wide range of UV absorption [21,44]. For better understanding of the photosensitivity of the BDK-doped PMMA, UV absorption properties of both pure PMMA and BDK-doped PMMA were investigated [19], as illustrated in Figure 1a. Compared to PMMA, two absorption bands appear in the BDK-doped PMMA system, with central wavelengths at ~250 nm and ~344 nm due to the strong $\pi -\pi^{*}$ transition and weak $n-\pi^{*}$ transition of BDK, respectively [45]. For the latter transition, due to an absorption at 355 nm, seen from the inset of Figure 1a, FBGs were successfully inscribed in the BDK-doped PMMA POF via a 355 nm UV laser inscription system [19].

In 2021, Hu et al., achieved a large refractive index change 6.2 × 10^−4^ during FBG inscription in BDK-doped PMMA POFs with a 266 nm UV laser [20]. The irritation wavelength of this laser is very close to the ~250 nm absorption peak, where the absorption is ~26 times as high as pure PMMA. The large increase of refractive index could be induced by a molecular orbit transition with free radical generation when exposed to UV light [19,20,21], as depicted in Figure 1b. Thus, large refractive index variations could result from the formation of benzil molecules from benzoyl radicals, as depicted in Figure 1c [21]. Additionally, the side chains of PMMA could be substituted by the initiating radicals after UV irradiation, which also could lead to a large refractive index increase 

### 2.2. TS

**Figure 2:**
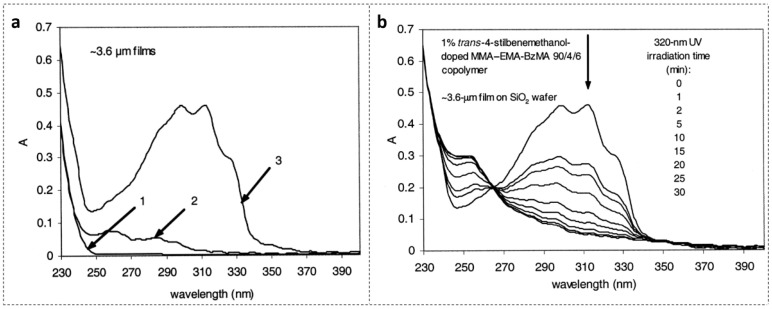
(**a**) UV absorption spectra: 1, cladding MMA-BMA (90/10 molar ratio); 2, core MMA-EMA-BzMA (90/4/6 molar ratio); 3, core TS-doped (1% wt) MMA-EMA-BzMA (90/4/6 molar ratio). Reprinted with permission from [27]. (**b**) Spectral change of TS-doped core with 320 nm UV irradiation versus time. Reprinted with permission from [27].

TS is a kind of stilbene derivative compound which exhibits a reversible *trans-cis* photoisomerization [46]. So, it has two structures, i.e., *trans* and *cis*. The refractive indices of both chemical isomers are different when they feature *trans* or *cis* structures [47]. Figure 2a shows UV absorption spectra of three types of materials in layers with a depth of ~3.6 μm [27]. Curve 1 shows the spectrum of the cladding material, methyl methacrylate (MMA)-butyl methacrylate (BMA) (90/10 molar ratio) copolymer, which is similar to the spectrum of PMMA. Curve 2 depicts the spectrum of core material without doping TS, MMA-ethyl methacrylate (EMA)-benzyl methacrylate (BzMA) (90/4/6 molar ratio) copolymer. Curve 3 exhibits the UV absorption spectrum of the TS-doped core material, MMA-EMA-BzMA (90/4/6 molar ratio) copolymer. There are three absorption peaks, centered at 298 nm, 310 nm, and 324 nm, respectively, for the TS-doped core material [26].

**Figure 3:**
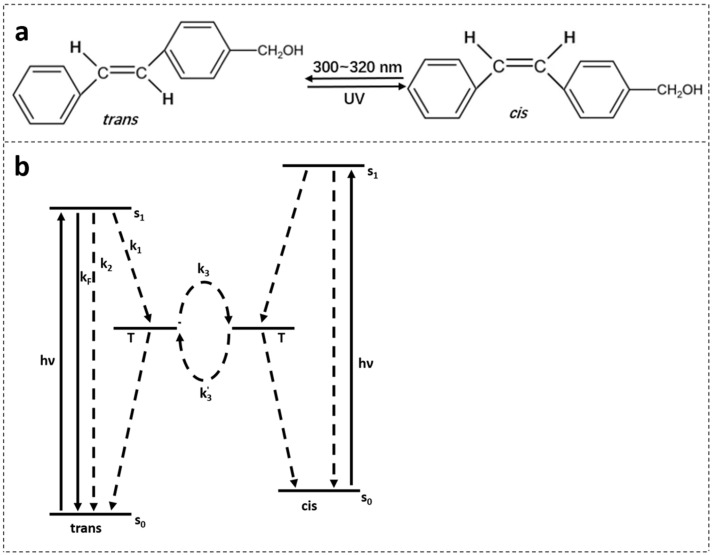
(**a**) 4-stilbenemethanol isomerization from *trans* to *cis* under UV irradiation [27,48]. (**b**) Energy level scheme involved in the photoisomerization process of the TS compound. Reprinted with permission from [29].

Figure 2b shows the spectral evolution with respect to different irradiation times in the *trans-cis* photoisomerization process [27,48]. Absorption peaks decrease with increasing irradiation time. Generally, *cis* isomers of stilbene and its derivatives have a relatively low absorption around the peak absorption wavelength of their counterpart *trans* isomers [27]. Figure 3a shows the photochemical transformation process of 4-stilbenemethanol from *trans* to *cis* structure under UV illumination [27,48], and the *trans*-isomer has a higher refractive index compared to *cis*-isomer due to the change in molecular polarity [46].

Under certain circumstances, *trans-cis* photoisomerization continues after UV irradiation [49], or a reverse process (*cis-trans* photoisomerization) occurs [29,49]. These effects could be attributed to the equilibrium between both intermediate states T in the scheme of energy levels involved in the photoisomerization process [50], as displayed in Figure 3b.

### 2.3. DPDS

**Figure 4:**
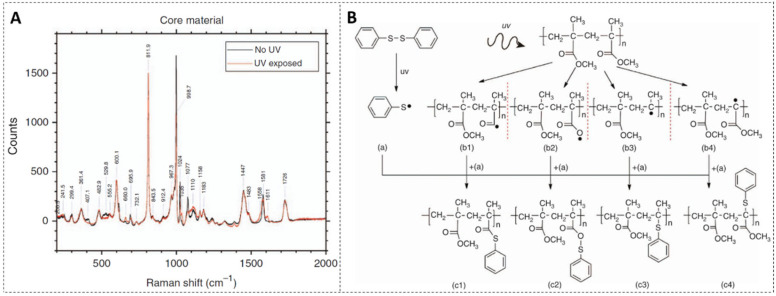
(**A**) Raman spectra before and after UV exposure on the DPDS-doped core material. Reprinted from [4]. (**B**) DPDS is split into two sulphenyl radicals upon UV exposure (**a**), four photodegradation reactions of the PMMA chain are considered, which are the removal of the: $OCH_{3}$ group (**b1**) and $CH_{3}$ group (**b2**) from the side chain, complete side chain (**b3**) and $CH_{3}$ group on the other side of the side chain (**b4**), followed by the attachment of the sulphenyl radical (**c1**–**c4**), respectively. Reprinted from [4].

DPDS is a kind of novel dopant, which contains two benzene rings connected by a disulfur bond. It can increase both the photosensitivity and the refractive index of the fiber core, which arises from photodissociation of the DPDS molecule and the following molecular reorganization. The refractive index change mechanism of DPDS-doped PMMA POF was investigated by Raman spectroscopy and electronic structure calculations [4]. First, the Raman spectra of the DPDS-doped core material before and after exposure to UV were compared, as shown in Figure 4A, presenting that both the changes in the carbon-sulphur bonding and the changes to the phenyl rings in DPDS after UV exposure can generate the change of refractive index of the fiber core [4]

Then, to further investigate the variation of DPDS when exposed to UV, a theoretical electronic structure calculation model was used. Under UV irradiation, the DPDS molecule is split into two sulphenyl radicals. This process is depicted in Figure 4B(a). Owing to the damage to the side chains of PMMA after UV exposure [51,52], various possible photodegradation effects were considered on a simulative PMMA chain consisting of two monomer units. Thus, various possible reaction pathways were considered to determine which reaction was most thermo-dynamically favorable [4], as shown in Figure 4B. Considering chemical reactions based on the energy calculations, four possible photodegradation reactions of the PMMA chain are presented (b1–b4), which are the removal of the $OCH_{3}$ group from the side chain (b1), the removal of the $CH_{3}$ group on the side chain (b2), the complete removal of the side chain (b3), and the removal of the $CH_{3}$ group opposite the side chain (b4). By comparing different reaction pathways, the attachment of the sulphenyl radical to the side chain after $OCH_{3}$ chemical group removal is the most energetically favorable, which agrees with the result of the changes shown via Raman spectroscopy [4].

### 2.4. 9-VA

**Figure 5:**
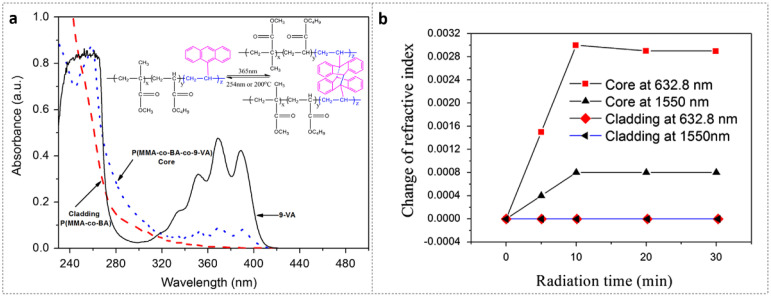
(**a**) Absorption spectra of cladding material [P(MMA-co-BA)] (dashed line), core material [P(MMA-co-BA-co-9-VA)] (dotted line) and pure 9-VA (solid line); The inset presents the reaction of photo-crosslinking and dissociation under 365 nm UV irradiation. Reprinted with permission from [30]. (**b**) The refractive index changes of core and cladding materials under 365 nm UV irradiation as a function of time. Reprinted with permission from [30].

9-VA is a derivative of anthracene and a typical photosensitive dye [53,54,55,56]. By doping photosensitive material 9-VA, both the photosensitivity and the stability of the POF can be enhanced [30]. The UV absorption spectra of pure 9-VA, core, and cladding materials are exhibited in Figure 5a. The solid line represents the pure 9-VA UV absorption spectrum, with three absorption peaks centered at 351 nm, 369 nm, and 388 nm, respectively. The dashed line displays the spectrum of the cladding material, poly-(methyl methacrylate-co-butyl acrylate) [P(MMA-co-BA)] (77.5/22.5 molar ratio), which is similar to the spectrum of the pure PMMA. The spectrum of the poly-(methyl methacrylate-co-butyl acrylate-co-9-vinylanthracene) [P(MMA-co-BA-co-9-VA)] (77.3/22.5/0.2 molar ratio) copolymer core material is presented by the dotted line. A large absorption can be observed over a wide wavelength range of 340–400 nm because of the doping of the 9-VA, which enables the core material to be photosensitive thanks to the photo-crosslinking by the photodimerization of anthracene under irradiation at near UV/vis band (seen in the inset of Figure 5a) [56].

Furthermore, the refractive index increases were measured for both core and cladding materials with 365 nm UV radiation [30], as shown in Figure 5b. It was found that in the same exposure condition, the core material doped with 9-VA is photosensitive with large refractive index changes, while the cladding material without 9-VA is not photosensitive. There are two functional groups in 9-VA, i.e., C=C group and anthracene group. The former is thermally active, while the latter is optically active [53,54]. The reverse reaction of photodimerization occurs under the ~254 nm UV irradiation or heated up to 200 °C [55,56], which guarantees the stability of the 9-VA-doped PMMA POF.

### 2.5. MVK

**Figure 6:**
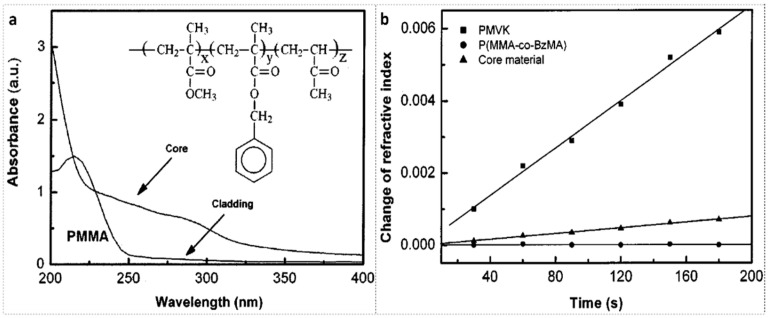
(**a**) Spectra of core material [P(MMA-co-MVK-co-BzMA)] and cladding material PMMA; Inset: chemical structure of core material. Reprinted with permission from [16]. (**b**) Refractive index changes of PMVK, P(MMA-co-BzMA) and core material under UV (wavelength over 300 nm) irradiation as a function of time. Reprinted with permission from [16].

MVK is a kind of photosensitive material, which could be doped in PMMA material as well. The film absorbance spectra of PMMA (cladding material) and P(MMA-co-MVK-co-BzMA) (core copolymer material) are shown in Figure 6a, and the chemical structure of the core material is shown in the inset of Figure 6a [16]. PMMA has negligible absorption beyond 250 nm, and the core material demonstrates considerable absorption in the wavelength range of 250–300 nm.

Figure 6b shows refractive index changes of PMVK, P(MMA-co-BzMA), and core material under UV (wavelength over 300 nm) irradiation as a function of time [16]. It is found that after 180 s of exposure, there is no detectable refractive index change for P(MMA-co-BzMA), but for PMVK and core material doped with MVK, the refractive index increases by ~0.0059 and ~0.0008, respectively. Thus, the refractive index change of the core material can be attributed to the UV photolysis of MVK to prepare graft copolymers [17].

### 2.6. AZO

**Figure 7:**
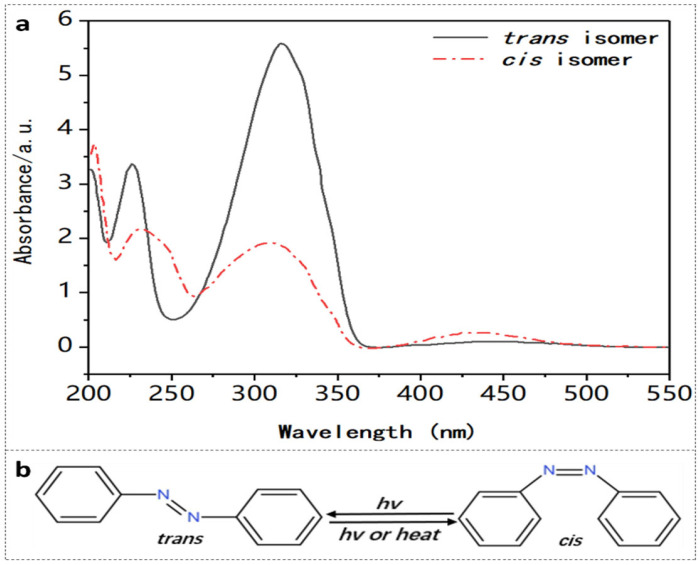
(**a**) UV-vis spectra of AZO before (*trans* isomer) and after (*cis* isomer) UV irradiation. Adapted with permission from [58]. (**b**) Schematic of AZO photoisomerization [57].

AZO has two different isomers, i.e., *trans*- and *cis*-AZO [57], which have different absorption spectra [58], as shown in Figure 7a. The absorption spectrum of AZO includes a strong and a weak absorption peak corresponding to the $\pi -\pi^{*}$ and $n-\pi^{*}$ electronic transition of the *trans*-isomer and the *cis*-isomer, respectively [59]. The *trans*-AZO presents a considerable absorption in the wavelength range of 320–380 nm owing to the *trans-cis* photoisomerization, while visible light irradiation in the range of 400–500 nm triggers the reverse *cis-trans* transition, accompanied by considerable heat generation [25,42,57]. The transformation process is depicted in Figure 7b. The absorption change on photoisomerization corresponds to the refractive index change, according to the Kramers–Kronig relationship [60].

In all cases, the *trans-cis* photoisomerization is reversible either by irradiation or by thermal excitation. In practice, the photo-induced refractive index change is relatively stable with time due to the slow thermal-driven *cis-trans* transition [59].

## 3. Photosensitive POF Fabrication Technique

It is well known that the drawing temperature for POFs is lower than silica optical fibers. Thus, all kinds of photosensitive organic dyes or copolymers can be doped into the POF to change the refractive index of the fiber core without being destroyed [61]. Moreover, dopants can greatly improve the photosensitivity of the material to reduce the grating writing time, improve the grating reflectivity, and even enable grating inscription in the fiber drawing process. Generally, a doped preform is prepared before fiber drawing. To achieve that, the tuning and controlling of the index are very important, ensuring that the refractive index of the core is a little bit higher than the cladding. One technique for doped preform fabrication is called the double-polymerization technique with two steps of polymerization [16,19,28,29,30,31,38,41,49,62,63,64]. First, a preform with a hollow core in the center is polymerized as a cladding preform. Then, a core with a dopant is polymerized inside the cladding preform to get a doped-core fiber preform. Another technique is called the pull-through technique [4,20,35,65], whereby a cane drawn from a doped core preform and a hollowed cladding preform are fabricated separately, and then a full preform is obtained by inserting the cane through the cladding preform. Additionally, following the diffusion doping technique, a preform or a fiber can be immersed in a solution mixed with photosensitive dopants, so that the dopant can diffuse into the material to enhance the photosensitivity of the fiber [33,34,36,42].

### 3.1. Double-Polymerization Technique

**Figure 8:**
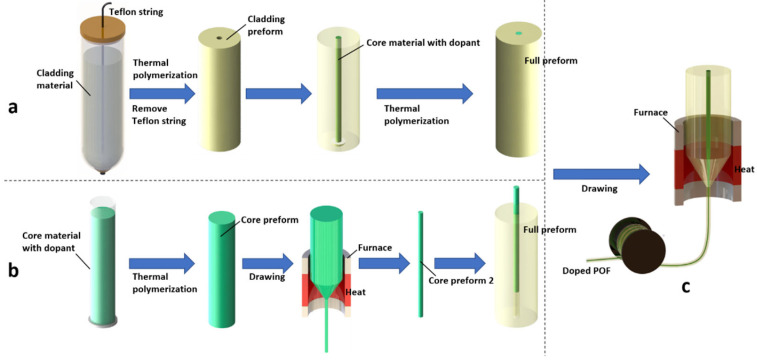
Schematic presentation of the fabrication processes of core-doped fibers using double-polymerization technique (**a**) [28,62] and pull-through technique (**b**) [4,35], and the fiber drawing facility (**c**).

Using the double-polymerization technique, the schematic of the fabrication process of a core-doped fiber preform is presented in Figure 8a. The fabrication process is mainly divided into two steps. In the first step, the cladding monomers are firstly imported into the tube with a Teflon string in the center, then fully polymerized by heat treatment to obtain a hollowed cladding preform after the removal of the Teflon string. In the second step, the hole is filled with core monomers and dopants, which are subsequently polymerized once more. As a result, a core-doped fiber preform is obtained. Finally, as shown in Figure 8c, the fiber preform is heat-drawn into a POF by a furnace and a taking up spool. One drawback of this technique is the diffusion of the core materials into the surrounding cladding during the core polymerization process, so that the core dimension increases, and it is difficult to obtain an SM fiber.

Peng et al. [62] first used the double-polymerization technique to achieve the fabrication of Rhodamine B-doped POFs in 1996. Then, in the work [19,31,64], the authors used this technique to make a BDK-doped PMMA POF. First, with a Teflon string, a hollow stick made of the copolymer of MMA-EMA was fabricated as a cladding preform. Then, the mixture of MMA, EMA, BzMA, BDK, initiator (lauryl peroxide), and chain-transfer agent (*n*-butyl mercaptan) were used as core materials, in which MMA was the base material. EMA and BzMA were used to adjust the refractive index, and BDK was used as the photosensitive material. Other than that, The Hong Kong Polytechnic University used the technique to produce TS-doped PMMA POFs [27,28,29,38,49]. In the work [27,28], the POF cladding was made by polymerizing MMA and BMA, and the core was made of a copolymer of TS-doped MMA-EMA-BzMA. In the work [29,38,49], the cladding of the POF was made of pure PMMA and the core was composed of PMMA doped with diphenyl sulfide (DPS) and TS. Here, DPS can facilitate the *trans-cis* interconversion of TS during UV irradiation process [66].

Using the same technique, the photosensitive POF with P(MMA-co-BA-co-9-VA) as a POF core material and P(MMA-co-BA) as a POF cladding material was prepared [30]. In addition, Luo et al., finished the fabrication of AZO-doped PMMA POF [41], which used azo-bis-isobutyronitrile as an initiator. In 2005, Li et al. [16] fabricated an MVK-doped PMMA POF using the technique. They used P(MMA-co-BA) as the cladding material, and MMA, BzMA, initiator (benzyl peroxide), chain-transfer agent (*n*-butyl mercaptan), and dopant MVK as the core materials. In the work [16,41], all the thermal polymerization processes were carried out in an oven filled with $N_{2}$ at a certain pressure. In addition to the fabrication of the core doped POF with this technique, the POF with a doped cladding could also be fabricated by the polymerization of the cladding preform. In 2014, Kowal et al., prepared a TS-doped cladding mPOF preform [36]. First, the inner preform made of pure PMMA with an arrangement of holes was fabricated, and then the outer doped cladding composed of MMA, TS, initiator (benzyl peroxide), and moderator (thioglicolid acid) was thermally polymerized surrounding the inner preform.

### 3.2. Pull-Through Technique

This POF fabrication technique was first presented by a team from The Hong Kong Polytechnic University [4]. Different from the double-polymerization technique, in which the core monomers are poured directly into the central hole of the cladding preform and then polymerized, in the pull-through technique, the core preform is prepared separately, avoiding the diffusion of the core materials into the surrounding cladding in the double-polymerization technique. The detailed fabrication process is depicted in Figure 8b.

For the first time in 2018, Bonefacino et al. [4] produced a DPDS-doped PMMA POF (pure PMMA as cladding material and DPDS-doped PMMA as core material) using the pull-through technique. Before the polymerization of the cladding, a pre-polymerization process to prepare the solution took place in a sealed glove box to avoid the oxygen reaction with the heated solution, and this procedure allowed fabricating preforms with fewer dust particles and lower humidity [4]. Afterwards, the cladding preform was fully polymerized by heat treatment. Separately, the core mixture is poured into a smaller test tube. After curing in the oven, the core preform was drawn into a cane with smaller dimensions using a custom-made POF drawing tower. Then, the cane was tightly inserted into the cladding preform to get the full preform. The resultant preform was drawn into the fiber, as depicted in Figure 8c. In the work [20,35,65], their team again successfully fabricated the BDK-doped PMMA POF by using this technique. The cladding of the POF is made of pure PMMA, and the core material consists of BDK, BzMA, and PMMA.

### 3.3. Diffusion Doping Technique

**Figure 9:**
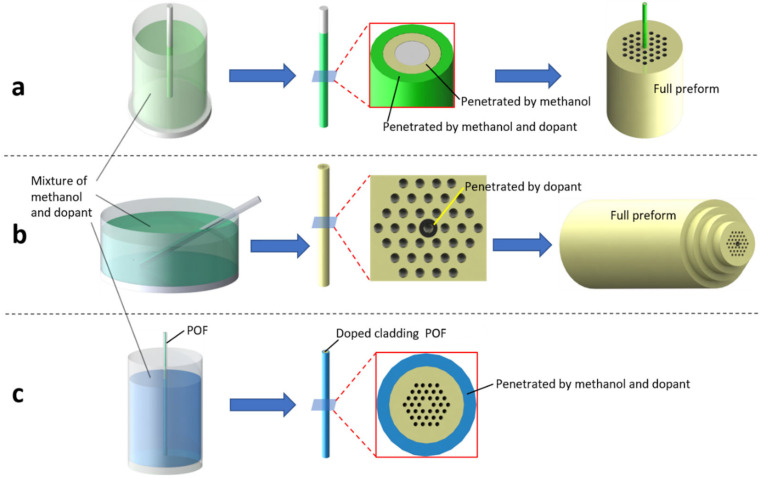
The fabrication process of doped mPOF by using diffusion doping technique: core preform diffusion (**a**), central hole diffusion (**b**), POF cladding diffusion (**c**) [33,34,42].

The diffusion doping technique mainly uses methanol to carry dopants into preform or fibers made of PMMA, in order to obtain a doped preform [33,34] or POF [42]. In the work [33], Sáez-Rodríguez et al., used this technique to fabricate the core-doped mPOF preform. The process is illustrated in Figure 9a. Firstly, by drawing a 60 mm diameter commercial PMMA rod, a 5.23 mm diameter unstructured solid cane was produced, which was then immersed in a solution of methanol and BDK for nine days at room temperature. Methanol works as a diffusion enhancer inside PMMA, because it effectively increases the migration rate of BDK in PMMA, thereby increasing the penetration of BDK [67]. The rod was then removed from the solution and the diffused methanol-BDK solution can be seen from the cross-sectional image of the cane. Two concentric colored circles indicate that the rod was not immersed in the solution long enough for the BDK to reach the center of the rod and methanal diffused faster than BDK. In the second step, the BDK-doped cane was inserted into the central hole of a cladding preform with three-ring hexagonal structure. Finally, the mPOF with a BDK-doped core was fabricated by drawing the preform.

In 2017, Hu et al., successfully fabricated a BDK-doped PMMA mPOF by using the selected center hole diffusion doping technique [34]. The process of fabrication is demonstrated in Figure 9b with several steps. First, a preform with three-ring hexagonal cladding structure and a central hole was fabricated by drilling in a commercial PMMA rod. Then, the preform was drawn to form a 5.5 mm diameter cane with 300 μm circular holes. Afterwards, the cane was immersed in a solution of methanol and BDK, but only the central hole was filled by capillary force, as other holes were blocked by UV glue. Then, the set-up was sealed and put into an oven at 51.5 °C for ~30 min to speed up the diffusion of BDK inside the center hole [68]. From the cross-sectional image of the cane, it is found that the center hole remained after the diffusion of BDK (black circle). Finally, the doped cane was positioned within three concentric tubes made of PMMA to form a new preform. The central hole was collapsed during the drawing process, and finally the BDK-doped PMMA mPOF was obtained.

In 2015, Kowal et al. [42] used the diffusion doping technique to achieve cladding-doped PMMA mPOFs. The fabrication process is shown in in Figure 9c. They doped AZO into the outer layer of the mPOF cladding by immersing the fiber in a solution of methanol and AZO to increase UV absorption for the quick inscription of LPFGs. The thickness of the AZO-doped layer in the PMMA mPOF was controlled by the diffusion time.

### 3.4. Doping Technique Comparison

Three different doped POF fabrication techniques have their own advantages and disadvantages. The double-polymerization technique prepares POF preforms by successively polymerizing cladding and core materials, which is convenient to adjust the concentration of the dopant in the core preform. However, in the process of core polymerization, there is a problem in that the core material diffuses into the cladding preform, which increases the core dimension. The pull-through technique can effectively avoid the diffusion problem by fabricating the core preform and the cladding preform separately, which is widely applied in the fabrication of an SM doped POF. Nevertheless, the technique requires two drawing processes, which increases the fabrication complexity. Compared with the previous two techniques, the diffusion doping technique is simpler, and it is easy to achieve cladding doping, but it is difficult to achieve the accurate adjustment of dopant concentration. For the three techniques, the polymerization temperature of the preform is normally about 40~120 °C, and the exact temperature depends on both the material and the polymerization time of the preform [4,16,41,64]. The temperature in the drawing process is usually about 110~225 °C, and the actual temperature results from both the speed and the stress during the drawing process [4,16,41,64].

## 4. Types of POF Gratings

### 4.1. Short Period Gratings

Usually, short period gratings have grating periods of less than 1 μm, and are also known as Bragg gratings. Specifically, according to different structures, short-period gratings are mainly divided into standard fiber FBGs, TFBGs, PSFBGs, and CFBGs.

#### 4.1.1. Standard FBGs

**Figure 10:**
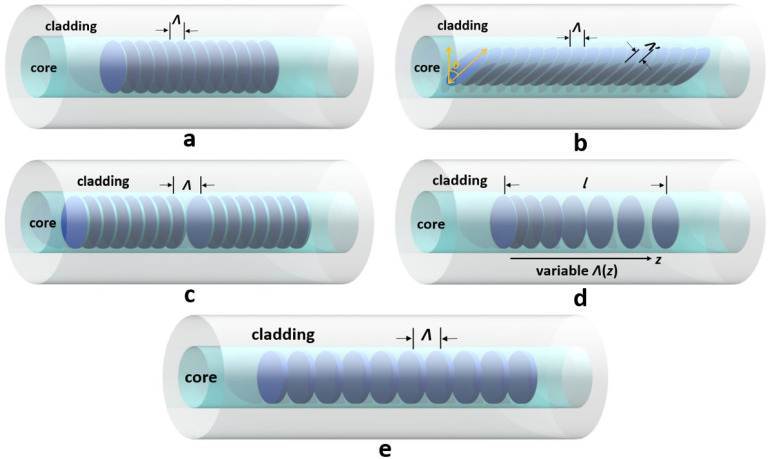
The structural diagrams of standard FBG (**a**), TFBG (**b**), PSFBG (**c**), CFBG (**d**) and LPFG (**e**).

FBGs are devices composed of a periodic modulation of refractive index in the core of an optical fiber, whose structural diagram is shown in Figure 10a. FBGs are commonly manufactured with the help of a laser by writing a periodic variation in the refractive index on the core of the fiber for a short length close to 1 cm [69,70].

Light guided along the fiber core is scattered by each grating plane. If the Bragg condition is unsatisfied, the reflected light from each of the subsequent planes becomes progressively out of phase and finally cancels out. Oppositely, if the Bragg condition is satisfied, the contributions of reflected light from each grating plane are added up in a constructive manner in the backward direction to form a back-reflected peak with a center wavelength defined by the grating parameters [69]. Such a grating works as a special mirror that reflects light only in a narrow band around the Bragg wavelength $\lambda_{Bragg}$, while transmitting other wavelengths. The Bragg wavelength is expressed by:

(1) $$\lambda_{Bragg}=2n_{eff,core}\Lambda$$

where $n_{eff,core}$ is the effective index of the core mode and Λ denotes the period of the index modulation along the fiber axis [69,70,71].

#### 4.1.2. TFBGs

TFBGs have a refractive index modulation direction slightly angled with respect to the optical fiber axis, whose spectrum is normally composed of a core mode and multiple cladding modes [37,72]. The TFBG structural diagram is shown in Figure 10b. The tilt angle θ determines Λ [73], as described by

(2) $$\Lambda =\frac{\Lambda^{\prime }}{\cos\theta }$$

where $\Lambda^{\prime }$ denotes the period of the interference pattern. The coupling efficiency and bandwidth of the extracted light are determined by the tilt of the grating planes and the modulation amplitude of the refractive index [69].

TFBGs have two mode-coupling mechanisms: self-backward coupling of the core mode and backward couplings between the core mode and numerous cladding modes [74,75]. The former generates Bragg mode or core mode similarly as FBGs, as expressed by Equation (1). For the latter coupling, TFBGs enable light to propagate in the cladding with different angles showing corresponding effective refractive indices of cladding modes $n_{eff,clad}$. Thus, the wavelength of the cladding mode resonance $\lambda_{clad}^{i}$ is in one-to-one correspondence with its effective refractive index. This relationship is expressed by the phase-matching condition:

(3) $$\lambda_{clad}^{i}=\left(n_{eff,core}+n_{eff,\;clad}^{i}\right)\Lambda$$

where the superscript *i* denotes the mode number.

#### 4.1.3. PSFBGs

A PSFBG consists of a grating where a discrete phase shift is inserted at a certain point inside the grating [76], acting as an in-fiber optical filter that transmits an extremely narrow linewidth within a Bragg grating stop band in a much broader reflected and transmitted spectrum. The structural diagram is shown in Figure 10c.

The phase shift φ between the rays reflected by the two Bragg gratings separated by the cavity can be estimated [77,78] by:

(4) $$\varphi =\frac{2\pi }{\lambda_{B}}n_{eff}2L$$

where L is the length of the introduced cavity.

#### 4.1.4. CFBGs

A CFBG, also called an aperiodic Bragg grating, is a grating that has a monotonically varying grating period along the fiber axis, which can be obtained by axially altering either the period Λ(z) of the grating or the index of refraction of the core, or both [70,79], so that the central wavelength of the Bragg mode λ(z) varies and each part of the grating reflects independently light with different wavelengths [80]. The most common CFBGs are gratings with linear variable period Λ(z) along the longitudinal extension in z-axis [81], whose structural diagram is shown in Figure 10d. Different Bragg wavelengths λ(z) are obtained along the fiber z-axis [79,82], according to:

(5) $$\lambda \left(z\right)=2n_{eff}\Lambda \left(z\right)$$

where Λ(z) is expressed by:

(6) Λ(z)=Λ(0)+kz

Here, l represents the grating length with 0<z<l; k is the chirp coefficient, which defines the increase rate of refractive index modulation period.

Besides, a cascade of several gratings with increasing period can also be used to simulate CFBGs [69], which are usually fabricated by the phase mask technique.

### 4.2. LPFGs

A LPFG is a type of grating with a period of refractive index modulation (>100 μm) much greater than the wavelength of the light guided in the fiber core. The structural diagram of the LPFG is shown in Figure 10e. In an optical fiber, the LPFG couples the forward-propagating core mode and the cladding modes, where the light is lost owing to the absorption and scattering with multiple depressions in their transmitted spectrum [83,84,85,86,87], as described by the phase matching condition:

(7) $$\lambda_{clad}^{i}=\left(n_{eff,core}-n_{eff,clad}^{i}\right)\Lambda_{LPFG}$$

where $\Lambda_{LPFG}$ is the period of a LPFG [88].

## 5. Grating Inscriptions in Doped PMMA POFs

Since the first report in 1999 that FBGs were successfully photo-inscribed in POFs [8], the photo-inscription technology has been widely investigated to manufacture FBGs, TFBGs, PSFBGs, CFBGs, and LPFGs thanks to the high photosensitivity of PMMA POFs doped with highly photosensitive materials either in the cores or claddings.

### 5.1. FBGs in POFs

Among various types of gratings, FBGs are the most common ones. Many researchers have succeeded in inscribing the FBGs efficiently in POFs with photosensitive cores [4,19,20,23,27,28,29,31,32,33,34,38,43,49,71,89]. Among them, the BDK-doped core is the most popular, whose refractive index increases under UV irradiation [19,20,23,31,32,33,34,43]. Besides, the photosensitivity of the POF can be improved by doping TS [27,28,29,38,49,71,89], which is another popular photosensitive material.

**Figure 11:**
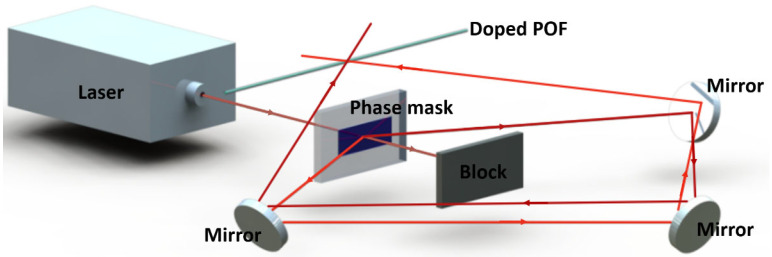
The diagram of inscription system based on Signac optical ring interference.

Normally, there are two systems for inscribing FBGs. The first inscription system is based on Signac optical ring interference [31,64,90], and only the two first-order beams diffracted by the phase mask are involved in the interference generation while blocking the zero-order diffraction beam [8], as shown in Figure 11. FBG inscriptions by the Signac optical ring interference system were reported by [19,31,32,64]. Luo et al., achieved the rapid fabrication of the FBG with a reflection peak up to 14.4 dB for only 4 min on a BDK-doped multimode (MM) PMMA POF using a 355 nm Nd:YAG pulse laser in 2012 [32].

**Figure 12:**
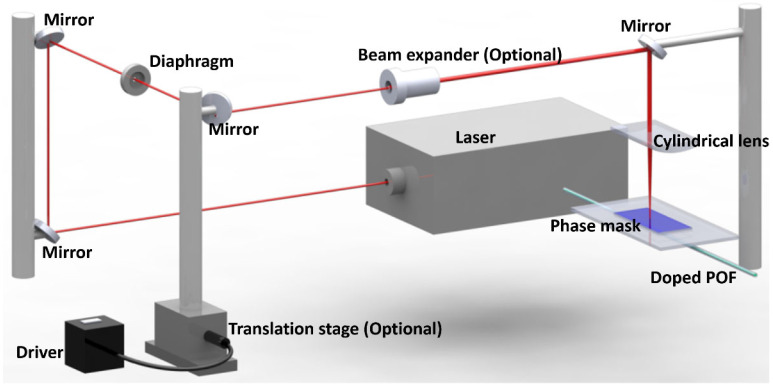
The diagram of inscription system based on laser and phase mask technique.

Additionally, researchers use the laser and phase mask technique to inscribe FBGs, which is more popular [23,38,43,71,89]. The set-up is illustrated in Figure 12. Here, the output laser beam is directed to the POF by four mirrors to maintain the output polarization state [89]. A diaphragm is used to shape the laser beam. An auto-driven translation stage is used to scan the laser beam along the fiber core for grating length improvement. A beam expander can also be used to increase the grating length. A cylindrical lens is hired to focus the laser beam to increase the power density on the fiber core. A phase mask optimized at laser emitting wavelength is positioned closely before the fiber to form interference light.

**Figure 13:**
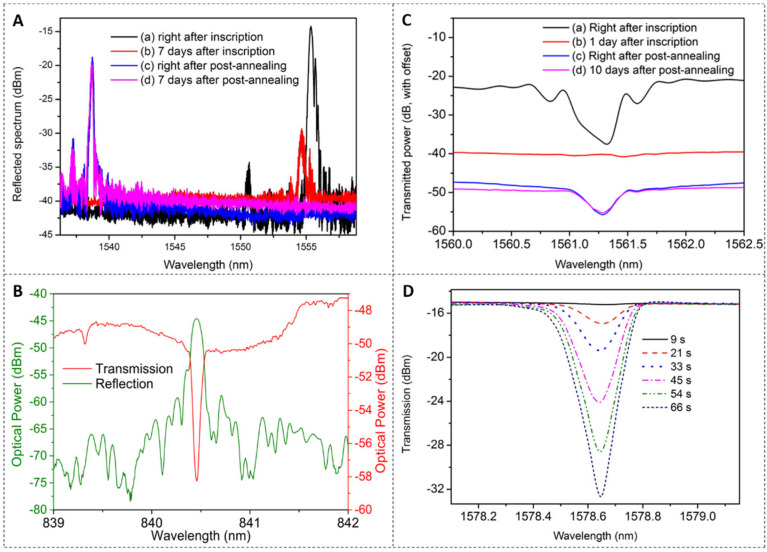
(**A**) Reflected amplitude spectra of FBGs [(**a**) just after inscription, (**b**) 7 days after inscription, (**c**) just after post-annealing, and (**d**) 7 days after post-annealing]. Reprinted with permission from [29]. (**B**) Reflection and transmitted amplitude spectra of the inscribed FBG. Reprinted from [43]. (**C**) Transmitted amplitude FBG spectra [(**a**) just after inscription, (**b**) 1 day after inscription, (**c**) just after post-annealing for 1 day, and (**d**) 10 days after post-annealing]. Reprinted with permission from [20]. (**D**) The evolution of transmitted amplitude spectra during the FBG inscription process. Reprinted with permission from [49].

For the laser and phase mask technique, different types of lasers were used as the light sources, such as the 325 nm CW He-Cd UV laser [4,29,33,35,71,89], 248 nm KrF UV pulse laser [23,38], 266 nm UV pulse laser [20,43], and 325 nm Sirah CSTR-6-28 pulsed dye laser [27,28]. Using a CW He-Cd laser, Sáez-Rodríguez et al. [33] manufactured FBGs with reflectivity of more than 99% in 13 min in BDK-doped PMMA mPOFs. With the same laser, Hu et al. [29] produced FBGs with a 25 dB reflection peak within only 1 s in a TS-doped SI PMMA POF, as shown in Figure 13A(a). However, the grating reflectivity decayed seven days after the inscription, as shown in Figure 13A(b). Thanks to a post-annealing process at 80 °C for two days, the grating spectrum recovered and remained after another seven days, as shown in Figure 13A(c,d), which could be attributed to the isomerization of 4-stilbenemethanol from *trans*- to *cis*- isomer under heating [29]. The wavelength shift of ∼16 nm could be attributed to the release of the frozen-in stress in the POF [91]. In addition, via the same laser, Bonefacino et al. [4] demonstrated the fabrication of FBGs with 14.8 dB reflection peak in 7 ms in the POF with a unique core dopant DPDS, and they added a 10× beam expander to expand the beam during the experiment.

Using a 248 nm KrF pulse laser, Pospori et al., reported an FBG with over 98.4% reflectivity on a BDK-doped PMMA POF with a single pulse ~15 ns [23]. Min et al. [38] inscribed an FBG with a ~17 dB reflection peak in a TS-doped photosensitive SI PMMA POF in only 0.4 s (40 pulses). Pereira et al. [43] reported an FBG (~84% reflectivity) inscribed in a BDK-doped PMMA mPOF with a single pulse ~8 ns from a 266 nm pulsed laser, whose reflected and transmitted amplitude spectra are shown in Figure 13B. With the same laser, Hu et al., achieved an FBG with a reflectivity of 97.1% on a BDK-doped SI PMMA POF using only one pulse [20]. Furthermore, the stability of the FBG was investigated, as shown in Figure 13C. The transmitted spectra of FBG just after inscription, as shown in Figure 13C(a), were not stable but with decay, and it almost vanished after one day, as depicted in Figure 13C(b). Similar to the work [29], the FBG was post-annealed at 80 °C for one day to recover, whose reflectivity returned to 78.3% and then almost remained after 10 days, as presented in Figure 13C(c,d).

In 2010, Tam et al., inscribed an FBG (87.4% reflectivity) for 10 min via a 325 nm Sirah CSTR-6-28 pulsed dye laser [28]. Besides, Hu et al., used a 400 nm femtosecond pulsed laser to fabricate an FBG (83% reflectivity) in only 40 s on BDK-doped PMMA mPOFs [34] and an FBG (98% reflectivity) on TS-doped SI PMMA POFs in ∼60 s [49]. The evolution of the transmitted amplitude spectrum of the FBG on the TS-doped PMMA POF during the photo-inscription process is illustrated in Figure 13D.The detailed FBG fabrication parameters in doped PMMA POFs are shown in Table 2.

### 5.2. TFBGs in POFs

**Figure 14:**
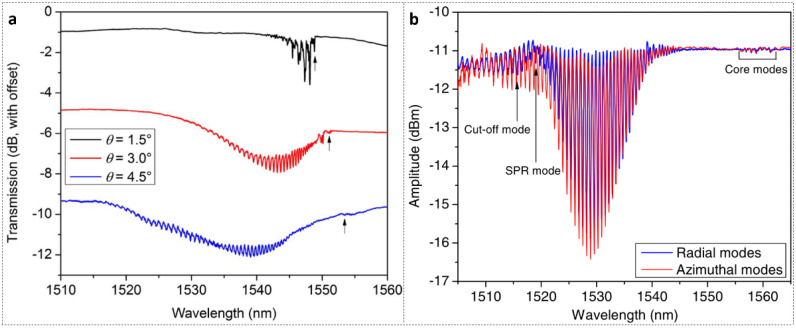
(**a**) Transmitted amplitude spectra of 6-mm-long TFBGs photo-inscribed at different tilt angles in slightly etched TS-doped POF. Reprinted with permission from [37]. (**b**) The transmitted amplitude spectra of 6° TFBG-SPR immersed in a solution with refractive index 1.408 in orthogonal polarizations. Reprinted with permission from [72].

In 2014, Hu et al. [37] first reported TFBGs photo-inscription at different tilt angles (1.5°, 3°, 4.5°) in etched TS-doped SI PMMA POFs, whose transmitted amplitude spectra are shown in Figure 14a. An arrow below each curve identifies the core mode resonance. The experimental set-up was similar to the work [89], with a phase mask slightly angled perpendicular to the optical fiber axis. The 3° TFBG was characterized by immersion in calibrated liquids with a refractometric sensitivity of ∼13 nm/RIU (refractive index unit). In the next year, with the same POF and inscription set-up as [37], Hu et al. [72] reported the first excitation of surface plasmon resonance (SPR) using a ~50 nm gold-coated 6° TFBG, whose transmitted amplitude spectra in orthogonal polarizations are shown in Figure 14b. The refractometric sensitivity reached more than 500 nm/RIU.

### 5.3. PSFBGs in POFs

**Figure 15:**
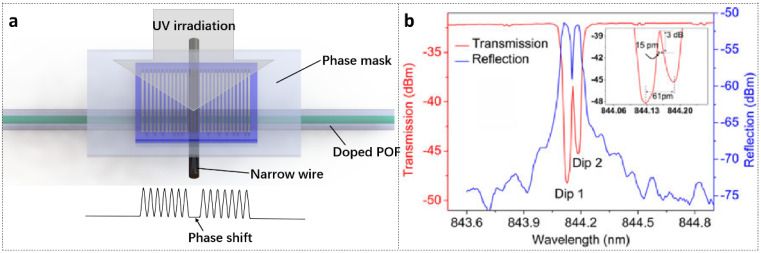
(**a**) Schematic configuration of PSFBG inscribed with a narrow metal wire positioned in the center of the UV beam. (**b**) Transmitted and reflected amplitude spectra of PSFBG inscribed by a single UV pulse. Reprinted with permission from [77].

In 2017, Pereira et al. [77] reported high-quality PSFBGs inscribed on SM BDK-doped PMMA POFs with a single 248 nm KrF laser pulse (width 15 ns) and phase mask technique. The phase shift was generated during the FBG inscription process by positioning a narrow metal wire with 40 μm in diameter as a blocking aperture near the phase mask in the center of the irradiation beam, as sketched in Figure 15a. This resulted in the inscription of two FBGs divided by a small gap in a single exposure with a phase shift on the structure. The transmitted and reflected amplitude spectra of the PSFBG are illustrated in Figure 15b with two strong dips in transmission, which were 16.3 dB (97.6% in reflectivity) and 13.2 dB (95.2% in reflectivity). The distance between the dips was ~61 pm, as shown in the inset of Figure 15b.

**Figure 16:**
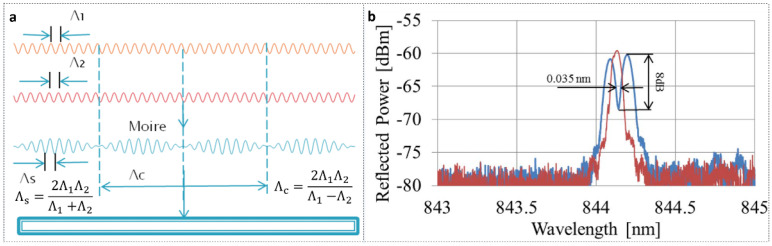
(**a**) Principle of Moiré grating: $\Lambda_{1}$ and $\Lambda_{2}$ represent the periods of grating 1 (orange line) and grating 2 (red line), respectively; $\Lambda_{s}$ and $\Lambda_{c}$ are obtained by superimposing grating 1 and grating 2 (blue line). Reprinted with permission from [76]. (**b**) Reflected amplitude spectra of first uniform FBG (red line) and FSFBG (blue line). Reprinted with permission from [76].

In 2018, Min et al. [76] demonstrated a simple methodology to manufacture PSFBGs in BDK-doped PMMA POFs using the phase mask technique. Two gratings with equal amplitude but different periods, $\Lambda_{1}$ and $\Lambda_{2}$, superimposed, as a Moiré grating [92]. This structure has two envelopes. One varies rapidly while the other varies slowly with period $\Lambda_{s}$ and $\Lambda_{c}$, respectively, as shown in Figure 16a. An intrinsic π phase shift appears at the crossover point. Here, each uniform FBG was inscribed by two UV pulses (width 15 ns) with a tiny grating period difference induced by different strains applied before inscriptions. Figure 16b shows the reflected spectra of the first uniform FBG and the FSFBG when two FBGs overlapped with a bandwidth of 0.035 nm for the 3-dB reflection band.

### 5.4. CFBGs in POFs

**Figure 17:**
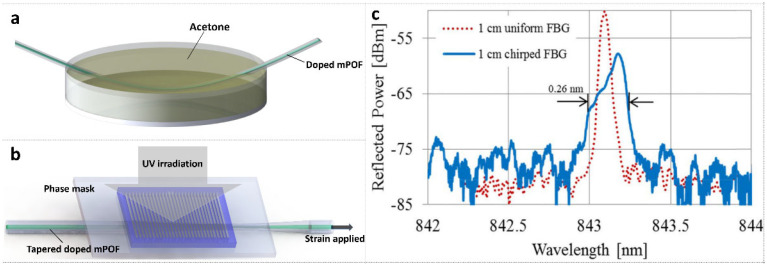
(**a**) Sketch of tapered mPOF fabrication. (**b**) CFBG inscription set-up with tapered mPOF and a uniform phase mask. (**c**) Reflected amplitude spectra of CFBG and uniform FBG. Reprinted from [93].

In 2018, Min et al. [93] demonstrated CFBG fabrication in BDK-doped tapered PMMA mPOF using a single KrF laser pulse and a uniform phase mask. Before inscription, the fiber was tilted and immersed in a container full of pure acetone to achieve a different radius along the fiber axis due to the volatile property of acetone, as depicted in Figure 17a. Then, a CFBG was inscribed in the tapered mPOF under 1% pre-strain induced by the stress-optic effect with strain gradient along the fiber, as shown in Figure 17b. The reflected amplitude spectrum of the CFBG with 0.26 nm chirp and the counterpart of a uniform FBG for comparison are shown in Figure 17c.

**Figure 18:**
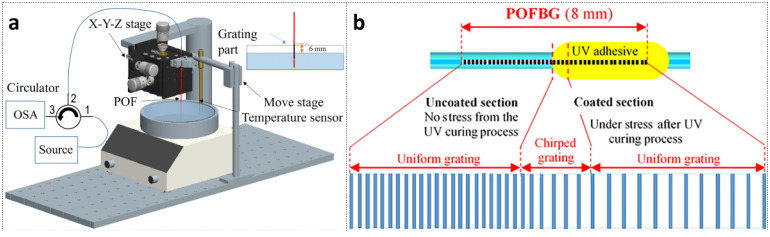
(**a**) The experimental set-up for gradient thermal annealing. Reprinted from [94]. (**b**) Sketch of the produced CFBG structure with period variation along the grating length after UV curing. Reprinted with permission from [95].

In the same year, Min et al., obtained CFBGs with 5.5 nm chirp [94] by applying hot water gradient thermal annealing for ~150 s to a 10-mm-long FBG inscribed in a BDK-doped PMMA mPOF. The proposed method is very simple requiring no special phase mask or additional etching procedure. The experimental set-up is shown in Figure 18a. A 4 mm part of the FBG was immersed in the water while the rest of the FBG was kept above the water.

In 2021, Min et al. [95] fabricated a CFBG with 11.7 nm chirp in a pre-strained 8-mm-long BDK-doped PMMA POF using UV-curing optical adhesive coated on half of the uniform FBG length. The cured adhesive maintained the grating pitch, while the grating pitch decreased in non-cured section when the strain was removed. Thus, there was a transition section with increasing grating pitch due to varied coating thickness along the fiber. Finally, a CFBG was obtained. A sketch of the produced CFBG structure is shown in Figure 18b.

### 5.5. LPFGs in POFs

**Figure 19:**
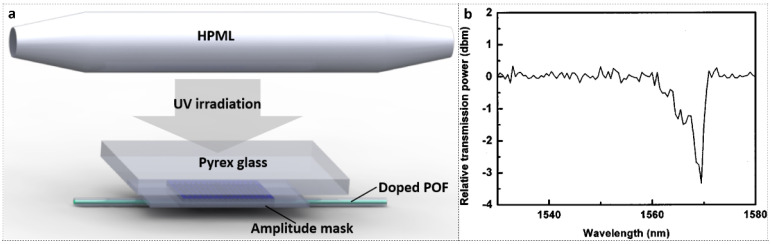
(**a**) Experimental set-up of LPFG inscription in an MVK-doped POF with an HPML; (**b**) The transmitted amplitude spectrum of the LPFG. Reprinted with permission from [16].

In 2005, Li et al., first successfully inscribed a LPFG in an MVK-doped PMMA POF using a high-pressure mercury lamp (HPML) and amplitude mask technique. A Pyrex filter was used to effectively block UV light in the wavelength range of 200–300 nm to reduce absorption on PMMA cladding. The experimental set-up is shown in Figure 19a [16]. After inscription, a ~3 dB resonant peak in transmission was obtained, as depicted in Figure 19b.

**Figure 20:**
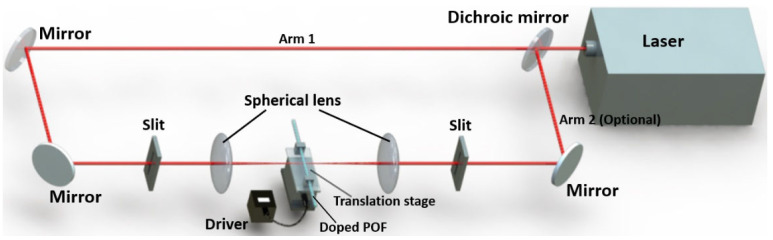
Experimental set-up for inscribing LPFGs in photosensitive POF [30,36].

In 2013, Wang et al., inscribed a LPFG with ~13 dB transmission loss in a 9-VA-doped PMMA POF under very low intensity (3.1 mW/cm^2^) for 10 min (2 min per point) exposure of UV lamp (365 nm dominant) [30]. The schematic of PbP inscription set-up is similar to Figure 20, but only one arm was employed in this work. Later, in 2014, Kowal et al., used the same technique to inscribe a ~20 dB LPFG in a TS-doped cladding PMMA POF, proving that the absorption enhancement in the outer doped layer shortened the overall fabrication time of the LPFGs by approximately six times compared to pure PMMA fibers [30,36]. Here, using a dichroic mirror, the beam from He-Cd laser was split equally into two beams. Symmetrical configuration of the inscription system reduced the fiber bending observed at the coupling points when the grating was fabricated using one side illumination.

In 2015, Kowal et al., used the same inscription technique to inscribe a LPFG with 15 dB resonance depth in a PMMA POF with a cladding doped with AZO [42]. In 2018, Min et al. [96] achieved a breakthrough in the efficiency of LPFG inscription with 2 s irradiation for one point, and a 20 dB LPFG was fabricated in a BDK-doped SM mPOF. The experimental set-up is similar to [30] with one side illumination. The detailed inscription of LPFG can be seen in Table 3.

## 6. Conclusions

In this paper, recent achievements in grating inscriptions in photosensitive POFs were summarized. Firstly, we reviewed the different photosensitive dopants, e.g., BDK, TS, DPDS, 9-VA, MVK, and AZO, and their refractive index change principles under UV irradiation. Secondly, in order to fabricate POFs doped with various photosensitive materials, either in cores or claddings, three different preform fabrication techniques were introduced, including the double-polymerization technique, pull-through technique, and diffusion doping technique. Thirdly, various types of gratings with different refractive index modulation structures were introduced, including standard FBGs, TFBGs, PSFBGs, CFBGs, and LPFGs. Finally, the fabrication of different types of gratings was presented. Thanks to the enhanced photosensitivity of the doped POF and phase mask technique, rapid, mass, and high-quality grating inscription can be obtained. Thus, less stability for grating inscription devices is required, and even gratings could be instantly inscribed in the process of fiber drawing. However, to date, as far as we know, most of the POFs for grating fabrication are homemade, which still need to be improved and industrialized for mass grating device fabrication and potential sensing applications.

## Figures and Tables

**Table 1:** The structure of dopants.

Dopant	Molecular Structure	
BDK	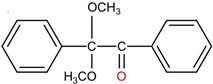	
TS	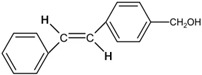	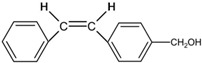
DPDS	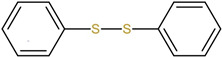	
9-VA	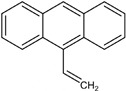	
MVK	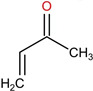	
AZO	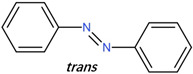	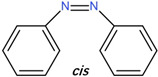

**Table 2:** The detailed FBG fabrication parameters in doped PMMA POFs.

Year	Fiber Type	Core Dopant	Laser	Phase Mask Period	Inscription Technique	Length	Fabrication Time	Reflectivity	Reference
2010	SM POF	BDK	355 nm Nd:YAG UV pulse laser	1061.4 nm	sagnac optical ring interference system	6 mm	17 min	reflection peak 6.7 dB	[19]
2011	SM POF	BDK	355 nm Nd:YAG UV pulse laser	1061.4 nm	sagnac optical ring interference system	-	16 min	reflection peak 5~8 dB	[31]
2013	mPOF	BDK	325 nm CW He-Cd UV laser	557.5 nm	scan phase mask technique	3.8 mm	13 min	99%	[33]
2010	SM POF	BDK	355 nm Nd:YAG UV pulse laser	1033.56 nm	sagnac optical ring interference system	6 mm	7 min	reflection peak ~7 dB	[64]
2012	MM POF	BDK	355 nm Nd:YAG UV pulse laser	1061.4 nm	sagnac optical ring interference system	6 mm	4 min	reflection peak 14.4 dB	[32]
2017	SM mPOF	BDK	248 nm KrF UV pulse laser	567.8 nm	phase mask technique	10 mm	4 min	98.4%	[23]
2017	SM mPOF	BDK	400 nm femtosecond pulsed laser	1060 nm	phase mask technique	10 mm	40 s	83%	[34]
2021	SM SI POF	BDK	266 nm DPS-266-Q pulse laser	1060 nm	phase mask technique	4 mm	1 pulse	97.1%	[20]
2018	SM SI mPOF	BDK	266 nm Nd:YAG UV pulse laser	567.8 nm	phase mask technique	8 mm	1 pulse	~84%	[43]
2019	SM SI POF	BDK	325 nm CW He-Cd UV laser	1046.3 nm	phase mask technique	10 mm	-	reflection peak ~25 dB	[35]
2004	SM POF	TS	325 nm Sirah CSTR-6-28 pulsed dye laser	1046.5 nm	scan phase mask technique	11 mm	~90 min	>98%	[27]
2014	SI POF	TS	325 nm CW He-Cd UV laser	1044 nm	scan phase mask technique	6 mm	40 min	>95%	[89]
2010	SM POF	TS	325 nm Sirah CSTR-6-28 pulsed dye laser	1046.5 nm	phase mask technique	-	10 min	87.4%	[28]
2006	SM SI mPOF	TS	325 nm CW He-Cd UV laser	1060.85 nm	phase mask technique	10 mm	8 min	reflection peak ~8 dB	[71]
2017	SI POF	TS	400 nm femtosecond pulsed laser	1060 nm	phase mask technique	10 mm	~60 s	98%	[49]
2016	SI POF	TS	325 nm CW He-Cd UV laser	1044 nm	phase mask technique	6 mm	1 s	reflection peak 25 dB	[29]
2018	SI POF	TS	248 nm KrF UV pulse laser	567.5 nm	phase mask technique	10 mm	0.4 s	reflection peak ~17 dB	[38]
2018	SM SI POF	DPDS	325 nm CW He-Cd UV laser	1046.3 nm	phase mask technique	-	7 ms	reflection peak 14.8 dB	[4]

**Table 3:** The detailed inscription of LPFG in doped POF.

Year	Fiber Type	Dopant	Laser	Period	Inscription Technique	Length	Fabrication Time	Resonance Amplitude	Reference
2006	SM POF	TS (core)	high-pressure mercury lamp (297 nm)	350 μm	amplitude mask method	-	40 min	-	[48]
2005	POF	MVK (core)	high-pressure mercury lamp	275 μm	amplitude mask method	3 cm	200 s	~3 dB	[16]
2013	POF	9-VA (core)	355 nm solid state laser	836 μm	PbP technique	40.1 mm	1 point (2 min)	~13 dB	[30]
2014	mPOF	TS (cladding)	325 nm CW He-Cd UV laser	1 mm	PbP technique	9 mm	1 point (42 s)	~20 dB	[36]
2015	mPOF	AZO (cladding)	325 nm CW He-Cd UV laser	1.2 mm	PbP technique	18 mm	1 point (20 s)	15 dB	[42]
2018	SM mPOF	BDK (core)	248 nm KrF UV pulse laser	1 mm	PbP technique	25 mm	1 point (2 s)	20 dB	[96]
